# Investigation of the Impact of Land-Use Distribution on PM_2.5_ in Weifang: Seasonal Variations

**DOI:** 10.3390/ijerph17145135

**Published:** 2020-07-16

**Authors:** Chengming Li, Kuo Zhang, Zhaoxin Dai, Zhaoting Ma, Xiaoli Liu

**Affiliations:** 1 Chinese Academy of Surveying and Mapping, Beijing 100830, China; cmli@casm.ac.cn (C.L.); ztmasm@163.com (Z.M.); liuxl@casm.ac.cn (X.L.); 2 College of Mining Engineering, North China University of Science and Technology, Qinhuangdao 063210, China; zhangkuocasm@163.com

**Keywords:** land-use distribution, PM_2.5_, boosted regression tree model, seasonal variations

## Abstract

As air pollution becomes highly focused in China, the accurate identification of its influencing factors is critical for achieving effective control and targeted environmental governance. Land-use distribution is one of the key factors affecting air quality, and research on the impact of land-use distribution on air pollution has drawn wide attention. However, considerable studies have mostly used linear regression models, which fail to capture the nonlinear effects of land-use distribution on PM_2.5_ (fine particulate matter with a diameter less than or equal to 2.5 microns) and to show how impacts on PM_2.5_ vary with land-use magnitudes. In addition, related studies have generally focused on annual analyses, ignoring the seasonal variability of the impact of land-use distribution on PM_2.5_, thus leading to possible estimation biases for PM_2.5_. This study was designed to address these issues and assess the impacts of land-use distribution on PM_2.5_ in Weifang, China. A machine learning statistical model, the boosted regression tree (BRT), was applied to measure nonlinear effects of land-use distribution on PM_2.5_, capture how land-use magnitude impacts PM_2.5_ across different seasons, and explore the policy implications for urban planning. The main conclusions are that the air quality will significantly improve with an increase in grassland and forest area, especially below 8% and 20%, respectively. When the distribution of construction land is greater than around 10%, the PM_2.5_ pollution can be seriously substantially increased with the increment of their areas. The impact of gardens and farmland presents seasonal characteristics. It is noted that as the weather becomes colder, the inhibitory effect of vegetation distribution on the PM_2.5_ concentration gradually decreases, while the positive impacts of artificial surface distributions, such as construction land and roads, are aggravated because leaves drop off in autumn (September–November) and winter (December–February). According to the findings of this study, it is recommended that Weifang should strengthen pollution control in winter, for instance, expand the coverage areas of evergreen vegetation like *Pinus bungeana* Zucc. and *Euonymus japonicus* Thunb, and increase the width and numbers of branches connecting different main roads. The findings also provide quantitative and optimal land-use planning and strategies to minimize PM_2.5_ pollution, referring to the status of regional urbanization and greening construction.

## 1. Introduction

With rapid socioeconomic development, new environmental problems represented by PM_2.5_ have appeared [1]. PM_2.5_ is airborne particulate matter with a diameter less than 2.5 μm, and it has strong adsorption characteristics. PM_2.5_ can directly enter human lungs through respiration, leading to various diseases, such as respiratory and cardiovascular diseases. It is a primary pollutant that influences atmospheric environmental quality, human health, and the Earth’s radiation balance [2,3]. From the perspective of geography, scientifically identifying the driving factors and impact mechanism of PM_2.5_ is critical for developing targeted PM_2.5_ pollution prevention and control strategies.

Many studies have been conducted on the relationships between PM_2.5_ pollution and its driving factors, such as assessing the correlations between PM_2.5_ and socioeconomic factors [4], meteorological factors [5], and land-use distribution [6,7]. Existing studies on the relationship between PM_2.5_ and land-use distribution have (1) mostly used linear regression models for analyzing the impacts of land-use distribution on PM_2.5_, ignoring the nonlinear effects of land-use factors on PM_2.5_. In addition, the research results are usually the fixed coefficients of impacts of land-use distribution on PM_2.5_ [8]. However, the relationships between land use and PM_2.5_ are complex, and with the variety of land-use factors, the impact on PM_2.5_ is not immutable; thus, existing methods cannot capture the heterogeneity in the impact value or provide guidance for the quantitative and optimal adjustment of land-use distribution. Additionally, these studies have (2) usually focused on annual-scale analysis, failing to capture seasonal differences in the impact of land-use distribution on PM_2.5_.

The above-mentioned shortcomings motivate our study. Based on PM_2.5_ data from 38 provincial monitoring stations in Weifang in 2017, this paper investigated the relationships between land use and PM_2.5_ in different seasons by using the boosted regression tree (BRT) model. The main objectives of this study were to (1) identify the distribution characteristics of PM_2.5_ and land-use types of Weifang; (2) measure the nonlinear effects of land-use distribution on PM_2.5_, capture how land-use magnitude impacts PM_2.5_, and investigate how the impacts vary across different seasons; and (3) explore the policy implications for urban planning to formulate quantitative pollution prevention and optimal land-use planning. This study helps to better understand the relationship between PM_2.5_ and the spatial distributions of land uses in Weifang. More importantly, it provides quantitative policy recommendations and guidance for environmental governance specific to different seasons and land-use planning on the micro scale.

The paper includes five sections. Section 2 mainly summarizes a literature review on the relationship between PM_2.5_ and land-use distributions. Section 3 describes the study area, data sources, and methodologies. The results of the analysis of the relationship between land-use distributions and PM_2.5_ in different seasons, as well as policy recommendations, are presented in Section 4, and the conclusions are provided in Section 5.

## 2. Literature Review

There is an extensive collection of literature on the relationship between land-use distribution and PM_2.5_. For instance, Lu et al. (2018) analyzed the impact of land-use distribution on PM_2.5_ concentration in the Yangtze River Delta based on a linear regression model and showed that PM_2.5_ was closely related to land-use patterns. The spatial distributions of urban green spaces and construction lands have significant impacts on urban air pollution. Specifically, with the increasing of forest and grass area, significantly reducing PM_2.5_ concentration, and its correlation coefficients are −0.567 and −0.54, respectively, while it was significantly positively correlated with urban construction land, with a coefficient of 0.414 [9]. Lin et al. (2020) analyzed the relationship between land-use distribution and PM_2.5_ in Jiangsu Province based on a traditional regression model and reported that forest and industrial lands had greater impacts on PM_2.5_ concentration than other land-use types. More specifically, the distribution of industrial land significantly increased the concentration of PM_2.5_, while the distribution of forest effectively reduced the concentration of PM_2.5_ [10]. Lu et al. (2020) explored and analyzed the relationship between land-use distribution and PM_2.5_ concentration in China. This study concluded that the PM_2.5_ concentrations were higher on artificial surfaces, while farmland and desert land, and the coefficients of their influences on PM_2.5_, were all positive. Forest, grass, and unused land had lower PM_2.5_ concentrations, and their correlations with PM_2.5_ were all negative. This study suggested that the proportions of construction land, farmland, forest, and grass should be rationally coordinated to reduce the PM_2.5_ concentrations in China [11]. Łowicki, D. (2019) investigated the relationship between PM_2.5_ concentration and land-use distribution in Poland, and found that the green spaces distribution could effectively reduce the PM_2.5_ concentrations [12].

Additional studies have explored the correlation between PM_2.5_ concentration and land-use distribution by using a land-use regression model (LUR). Zhang et al. (2018) explored and analyzed the relationship between land use and PM_2.5_ concentration in China based on ground monitoring data and spatial econometric models and showed that the construction land and road length in the 1-km buffer were positively correlated with the PM_2.5_ concentration, while the grassland area and forest area in the buffer zone were negatively correlated with the PM_2.5_ concentration [13]. Yang et al. (2017) analyzed the impact of land-use distribution on PM_2.5_ in Nanchang city using a linear regression model and proved that land-use distribution had a significant effect on PM_2.5_ concentration. The length of roads and proportion of industrial lands within the 300 m buffer and the area of ecological lands in the 2.4 km buffer had the largest contributions to PM_2.5_, and the impact did not vary with season [14]. Wu et al. (2017) performed a fitting analysis on PM_2.5_ in Beijing based on the LUR model and found that the length of roads within the 750 m buffer and the number of restaurants and the vegetation coverage in the 1.75 km buffer were important factors influencing the PM_2.5_ concentration [15].

However, this previous research has limitations. First, most research has adopted traditional regression models to explore the impact of land-use distribution on PM_2.5_, and these models do not reflect the nonlinear effects of land-use distribution on PM_2.5_ [11,16]; furthermore, these models do not clarify how the land-use magnitude impacts PM_2.5_, leading to possible estimation biases. Second, most studies are usually performed on the annual scale, and there is no accounting for the differences in the impact of land-use distribution on PM_2.5_ across different seasons [12].

## 3. Data and Methodology

### 3.1. Study Area

**Figure 1:**
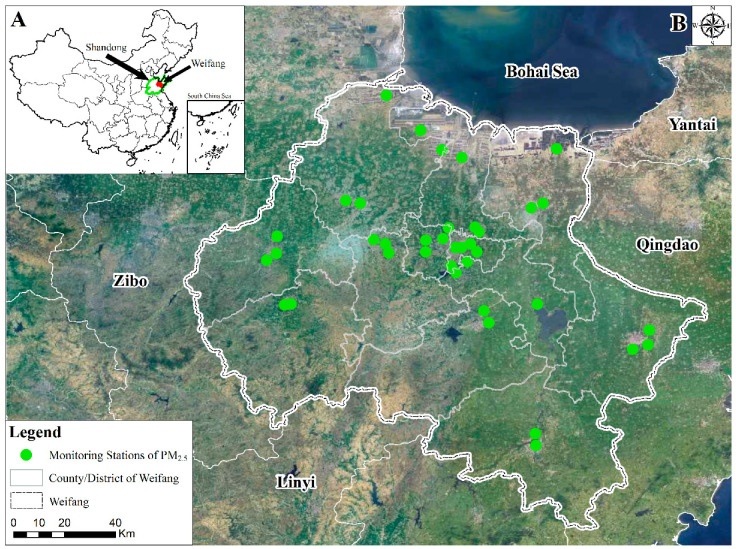
Study area and the spatial distribution of monitoring stations (**A**: the location of Weifang City; **B**: monitoring sites).

Weifang city (35°32′N–37°26′N, 118°10′E–120°01′E), located in the central Shandong Peninsula (Figure 1), is adjacent to Zibo in the west, Linyi in the south, Qingdao in the east, and Laizhou Bay and the Bohai Sea in the north. It contains four districts, six cities, and two counties, with a total area of 16,000 km^2^. The south is mainly covered with hills and low mountains, while the northeast is mainly characterized by plains and lakes [17]. Weifang city has four distinct seasons, with the ranges of the daily average temperature in spring (March–May), summer (June–August), autumn (September–November), and winter (December–February) are around 3–28 °C, 18–33 °C, 3–27 °C, and −6–6 °C, respectively. The range of relative humidity across the four seasons is around 50–60%, 60–80%, 50–75%, and 60–70%, respectively. As a regional comprehensive transportation hub, Weifang city has been experiencing industrial and economic advancements; however, the air pollution accompanying these advancements is intensifying. According to the comprehensive air quality ranking of 168 key Chinese cities issued by the Ministry of Ecology and Environment in 2019, Weifang was ranked moderately low [18,19]. The terrain and land types in Weifang are complex and diverse, and the spatial distribution of these land types has important effects on air pollution. However, there is no report on the relationship between land-use distribution and air quality in Weifang; therefore, it is urgent to conduct research on the correlation between PM_2.5_ and the spatial distribution of land use in Weifang.

### 3.2. Data Source

The PM_2.5_ monitoring station data used in this study were obtained from 5 national stations, 4 provincial stations, and 29 urban stations in Weifang in 2017. The data were acquired by automatic fixed air quality monitors through 24 h continuous monitoring. Thermo Fisher 1405F monitoring instruments were used to measure the PM_2.5_ concentrations, and this instrument operates on the principle of measuring PM_2.5_ concentrations by a filter dynamic measurement system (FDMS) with the tapered element oscillating microbalance (TEOM) [19,20]. The data collection frequency was 5 min, and the hourly and seasonal concentrations were obtained by calculating the arithmetic mean value of the data collected at intervals of 5 min every hour.

**Figure 2:**
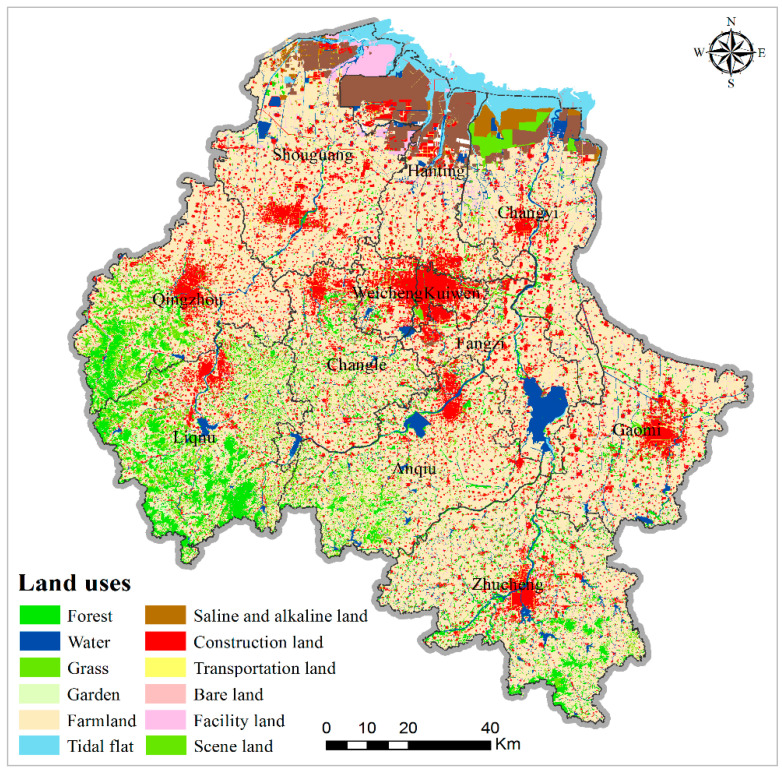
Spatial distributions of land uses in Weifang city.

Air pollution is closely related to the local land-use distribution at the micro scale; in particular, the spatial distribution of urban green space and construction land has significant impacts on urban air pollution. The land-use data in this study are from the Weifang Bureau of Natural Resources, which includes forest, grass, garden, farmland, water, construction land, unused land, transportation land, saline and alkaline land, facility land, bare land, scene land, and tidal flats (Figure 2). Eight widely distributed land-use factors (forest, grass, garden, farmland, water, construction land, unused land, and transportation land) were selected and their relationships with PM_2.5_ were assessed. To analyze the impact of transportation on PM_2.5_ in more detail, arterial road, secondary trunk road, and branch road data from OpenStreetMap were employed.

### 3.3. Methodology

#### 3.3.1. Data Integration and Hierarchical Grid Structure

**Figure 3:**
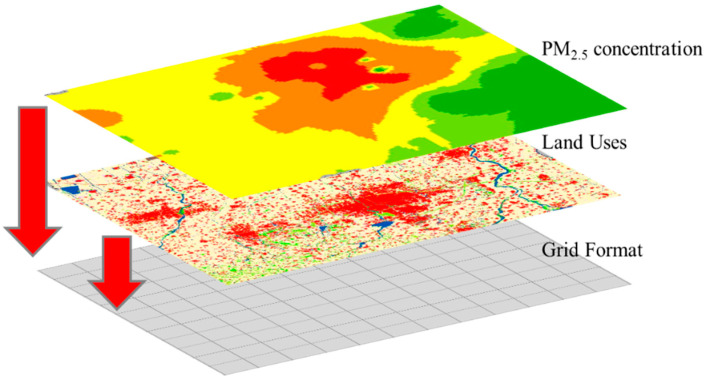
Data integration and grid structure.

Figure 3 illustrates the integration of the factors into this grid. First, based on 5 km grid cells, Weifang city was divided into uniform grids (Figure 3). Note that in this paper, different grid cells (1 km, 2 km, 3 km, 4 km, 5 km, 6 km, 7 km) were tested, and 5 km proved the best statistical fit with the highest cross validation accuracy. So, in order to better capture the relationships between land-use distribution and PM_2.5_, a grid 5 km cell was applied. Then, to obtain the land-use data and PM_2.5_ concentrations in every grid cell of Weifang, the grid cell was used to integrate the land-use data and PM_2.5_ concentration data (obtained by inverse distance weighting interpolation, IDW) [21]. It should be noted that the IDW method, one of the most commonly used interpolation methods, was applied in this paper due to the advantage of being simpler, intuitive, and more efficient when it comes to spatial interpolation [22]. The land-use data were summed on the basis of the 5 km grid cells. For the PM_2.5_ concentration data, the mask-based extraction method was used to obtain the average PM_2.5_ concentration in each grid cell.

#### 3.3.2. Boosted Regression Tree Model

In this paper, a machine learning statistical model, the boosted regression tree (BRT), was used to explore the quantitative impacts of land-use distributions on PM_2.5_ in Weifang. Existing studies have mostly used standard linear regression models to analyze the relationship between PM_2.5_ and land-use distribution. These models can capture the importance of each land-use factor and its effect on PM_2.5_ pollution by comparing regression coefficients. However, the impact coefficient of each factor is constant, which cannot reflect the complex nonlinear and dynamic relationship between PM_2.5_ and land-use distribution. This problem can be solved by introducing the BRT model [23].

The BRT model is a self-learning method based on the classification and regression tree (CART) algorithm, including the regression tree algorithm and the boosting method [24]. Recursive binary splitting in the algorithm is used to eliminate the interaction between various influencing factors, and the boosting method is used to establish a small regression tree set to express the nonlinear relationship with each influencing factor. The BRT model is an iterative process in which tree-based models were fitted iteratively using recursive binary splits to identify poorly modeled observations in existing trees until a minimum model deviance was reached [25]. To improve the performance of the model and reduce overfitting, the BRT model generates multiple regression trees through random selection and self-learning and extracts a certain amount of random data to analyze the impact degree of independent variables on dependent variables. The remaining data are used to test the fitting results. In this way, the stability and prediction accuracy of the model can be significantly improved [25]. Elith et al. (2008) recommended that the performance of the BRT model is superior to most traditional modelling methods, such as GLM (generalized linear model), GAM (generalized additive model), and multivariate adaptive regression splines [24].

In addition, BRT can capture the marginal effects of the independent variables. These marginal effects show that the relative influences of the independent variables vary with changes in their magnitudes and are scaled to be expressed as percentages [26]. The relative importance of variables can be measured based on the number of times a variable is selected for splitting, weighted by the squared improvement to the model as a result of each split, and averaged over all trees [27]. This means that BRT can capture the influence of a variable on dependent variable when the other independent variables take the mean value or are unchanged. The relative influence (or contribution) of each variable is scaled so that the sum adds to 100, with higher numbers indicating a stronger influence on the response [26]. A relative influence value greater than 0 indicates that the factor is positively correlated with the dependent variable, while a value less than 0 indicates a negative effect, and the value 0 indicates an insignificant correlation. The short upper mark in each figure delineates the successive 10% interval values for the land-use variable.

The BRT model has strong learning ability and flexibility when dealing with different data formats and complex data, and it does not need to consider the interaction and correlation between independent variables, which has an obvious advantage in studying the interaction between complex factors and performing forecast simulations. The BRT model has now been successfully applied in many fields, such as urban expansion, ecological modeling, and environmental science [25,28]. For instance, Yang et al. (2016) explored the impacts of environmental variables on soil organic carbon (SOC) and reported that the results assessed by the BRT model were greater than that of the RF (random forest) model [25]. In this study, the gbm package in the R language (Version 3.4.0, University of Auckland, Auckland, New Zealand) and the BRT package written by Elite (University of Melbourne, Parkville, Australia) were used to explore the seasonal variations in the impacts of land-use distribution on PM_2.5_ in Weifang. The BRT model is a supervised learning method, so the learning rate, tree complexity, and bagging fraction must be set during the training process. The learning rate means the contribution of each tree to the final fitted model, the tree complexity controls the size of trees and whether interactions between variables should be considered, and the bagging fraction specifies the proportion of data to be selected at each step [24].

## 4. Results and Discussion

### 4.1. Seasonal Variations in the Distribution of PM_2.5_

**Figure 4:**
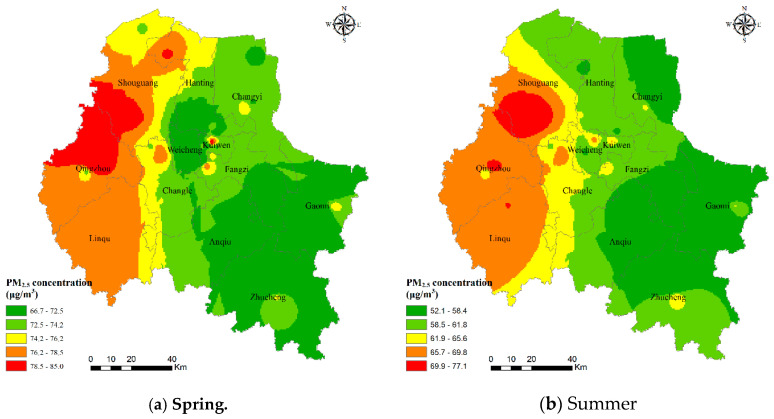

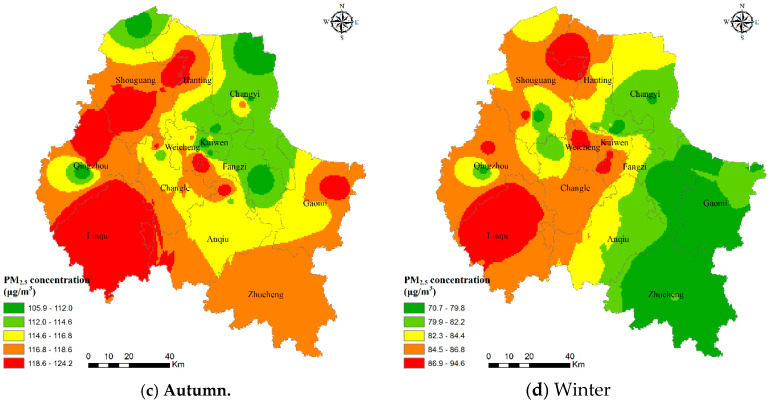
Spatial distribution of PM_2.5_ concentrations in the four seasons in Weifang.

Figure 4 shows the seasonal characteristics of the PM_2.5_ concentrations in Weifang city. Overall, the PM_2.5_ concentration shows a trend of being high in the west and low in the east of Weifang in 2017. For seasonal differences, Weifang has the highest PM_2.5_ level in winter, followed by that in autumn, and the lowest pollution level is in summer. The average PM_2.5_ concentrations in spring, summer, autumn, and winter are 74.09, 61.59, 83.33, and 116.10 μg/m^3^, respectively. PM_2.5_ pollution was more severe in Shouguang city, Qingzhou city, and Linqu County, which may be because there is much industrial land in the region and the affected areas are adjacent to Zibo city (an industrial city with more severe air pollution). It is noteworthy that the central area of Qingzhou showed an apparent ring-shaped low-value area, which was probably due to the distribution of numerous forests around this area.

### 4.2. Impact of Land-Use Distribution on PM_2.5_ across Different Seasons

The dependent variables are the averaged PM_2.5_ value in each 5-km grid cell, and the independent variables are the land-use factors. With a few combinations of BRT parameters tested, the final combination with minimum predictive deviance was determined. The learning rate, tree complexity, and bagging fraction were 0.005, 5, and 0.5, respectively. In each validation, 50% of the data were randomly selected for training, and 10-fold cross-validations were performed to select the best model. Using the BRT model, the impact characteristics and contribution rates of different land-use distributions on PM_2.5_ across different seasons were obtained. The correlations for the training data and cross-validation data in the four seasons were all greater than 0.80 (spring with 0.83, summer with 0.86, autumn with 0.81, winter with 0.80), indicating a good simulation verification effect.

#### 4.2.1. Explaining PM_2.5_ in Spring

**Figure 5:**
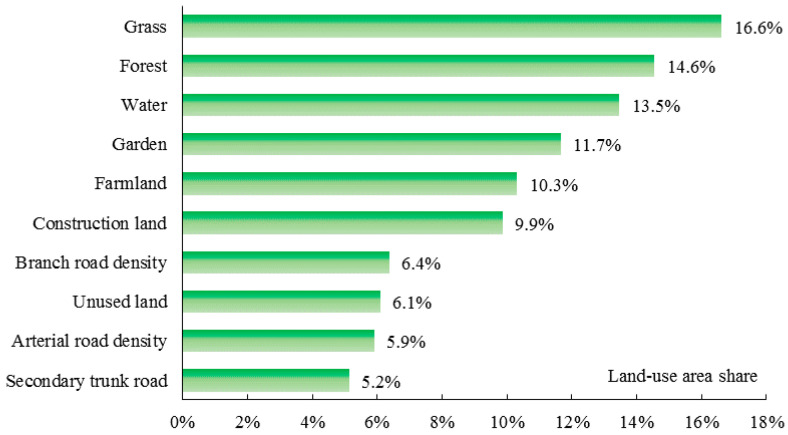
Relative impacts of each land-use distribution on PM_2.5_ in spring.

Figure 5 shows the contributing rates (%) that each land-use area share on PM_2.5_ in spring. The most important variable affecting PM_2.5_ in spring is the proportion of grassland area (16.6%), followed by the proportion of forest, which had a contribution rate of 14.6%. This result indicates that grassland has a stronger capability of adsorbing pollutants than forest in spring, which is probably due to slower growth of leaves in forest in early spring. Water, garden land, and farmland are also important for model predictions, with contributions of 13.5%, 11.7%, and 10.3%, respectively. Construction land has a relative influence of 10%, and the contributions of other factors vary between 5% and 10%.

**Figure 6:**
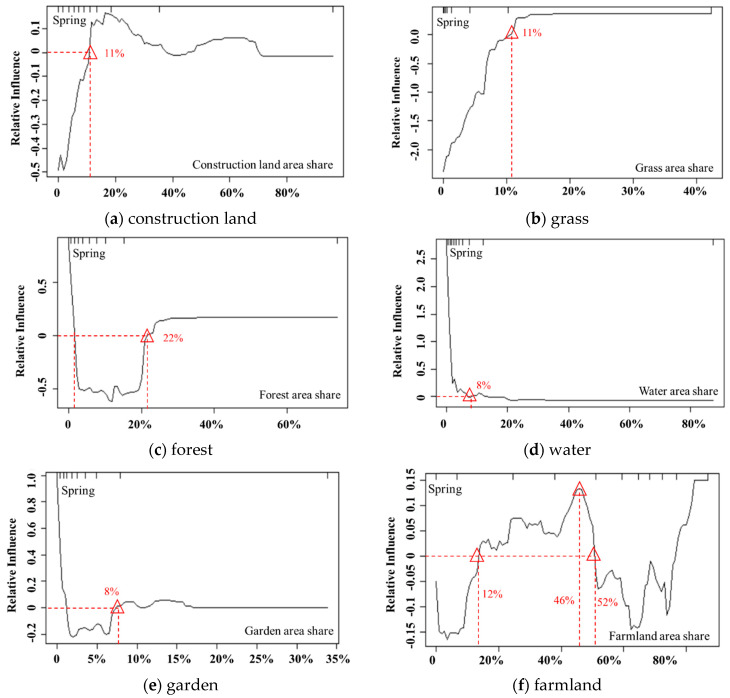
The influence of the main factors (concentration rate >10%) on PM_2.5_ in spring.

Figure 6 illustrates the impacts of land-use factors with a contribution rate greater than 10% on PM_2.5_ by showing how their relative influence on PM_2.5_ varies with changes in their magnitude.

The proportion of grassland area plays a significant role in alleviating PM_2.5_, especially when the proportion is less than 11%, indicating that grasslands can effectively adsorb pollutants to suppress PM_2.5_ pollution, as suggested by Zou et al. (2016) [29]. However, when the proportion of grassland area is greater than 11%, the impact curve tends to be planar, and the relative impact value is approximately 0, suggesting a non-statistically significant impact on PM_2.5_. The proportions of grassland area greater than 11% are only 10%, which can be ignored.

Forest has a strong inhibitory effect on PM_2.5_, and its relative influence displays a general downward trend overall, and its negative impact on PM_2.5_ concentrations increases and reaches a maximum at the threshold of 12%. Beyond this threshold, the impact slightly decreases and then remains constant at the threshold of 22%. It should be noted that when the proportion of forest area is less than 2%, the relative influence is greater than 0, which may be due to the slow growth of forest leaves and the weak adsorption capacity of air particles in smaller trees. However, shares below 2% are negligible because they only account for 10%.

Existing research has not yet drawn a definitive conclusion on the influence of water on PM_2.5_. Li et al. (2016) showed that water can effectively reduce PM_2.5_ concentrations because they are less subjective to human activities, industrial production, and exhaust emissions and have the capability of adsorbing atmospheric particulate matter [30]. However, Lu et al. (2020) reported that water bodies have a positive contribution to PM_2.5_ [11]. Our study showed a similar finding, especially for water bodies with smaller areas, and its positive effect on PM_2.5_ was significant. It is worth noting that from the perspective of pollutant discharge, water does not discharge pollution matter. However, some small-area waters, which tend to be scattered within other land uses, are greatly affected by the PM_2.5_ in the other land types due to air mobility, especially in areas with high pollution concentrations. Additionally, the evaporation of water renders high atmospheric humidity, which in turn makes the particulate matter deliquescent and poorly diffused, thereby aggravating PM_2.5_ pollution. With the increase in the water area, its aggravation effect on PM_2.5_ gradually decreases, and the impact value tends to be 0. This result is because large-area waters, such as the waters surrounding the Bohai Sea, are less affected by PM_2.5_ pollution in the other land uses; thus, the aggravation impacts on PM_2.5_ decrease.

Compared with grassland and forest, the inhibitory effect of gardens on PM_2.5_ was relatively small. Specifically, when the garden area share is between 1% and 8%, PM_2.5_ pollution is effectively alleviated. When the garden area share is greater than 8%, it has a weak positive correlation with PM_2.5_, but similar to grass and forest, the corresponding data are only 10%, indicating the amount is negligible. Compared with grassland and forest, the leaves of orchard trees cover a relatively small area, thus having a weak capability to adsorb air particulate matter. In addition, garden land requires fertilization and pesticide application, which may have a negative effect on air quality.

The influence of farmland on PM_2.5_ in spring alternates between positive and negative, which is closely related to the dual effects of farmland being a ‘source’ and ‘sink’ for PM_2.5_ [31]. In spring, some crops begin to be sown, and soil tilling, soil bareness, and straw burning all play a role as ‘pollution sources’ and significantly increase PM_2.5_ pollution. However, similar to other vegetation, farmland has played a role as a ‘sink’, and crop leaves can effectively adsorb air pollution; thus, with the increase in farmland area, its negative effects on PM_2.5_ exceed the positive effects at a threshold of 52%.

The impact of construction land on PM_2.5_ shows a fluctuating trend, which is different from the existing research that reported construction land had a positive impact on PM_2.5_ [32]. When the share of construction land area is less than 11%, construction lands have a negative effect on PM_2.5_; by analyzing the actual data, the reason is that these construction lands are mostly in rural areas and townships in southern Weifang, such as Taiping village in Zhucheng city and Dengjiazhuang village in Anqiu city, and the natural vegetation in these areas can effectively mitigate PM_2.5_. This indicates that when land-use share is small in a grid cell, its impacts on PM_2.5_ concentration may be easily affected by the discharge from surrounding landforms. As the area of construction land increases, construction land presents the strongest positive effects on PM_2.5_. It should be noted that the positive influence on PM_2.5_ first increased and then decreased, indicating the existence of the “environmental Kuznets curve”. When urbanization reaches a certain threshold (approximately 42% in Weifang), air pollution will decrease with increasing urbanization, which is different from the results of Tan et al. [33].

#### 4.2.2. Explaining PM_2.5_ in Summer

**Figure 7:**
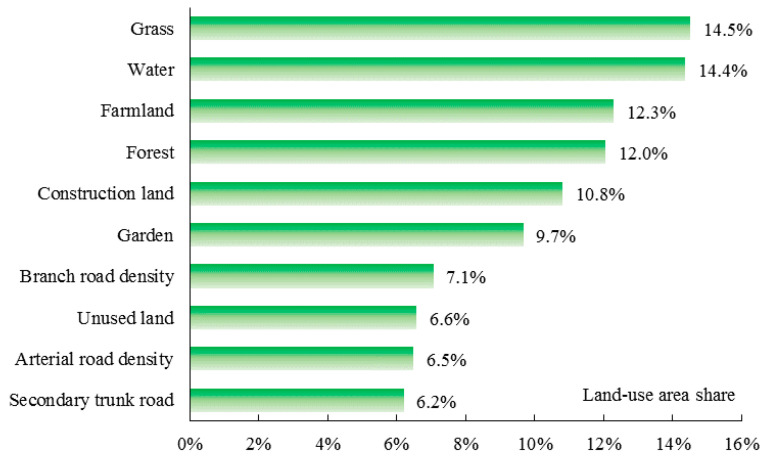
Relative impacts of each land-use distribution on PM_2.5_ in summer.

Figure 7 presents the contributing rates (%) of each land-use area share to PM_2.5_ in summer. Similar to spring, grassland is the dominant factor influencing PM_2.5_ in Weifang (14.5%). However, the impacts of water, farmland, and construction land on PM_2.5_ increase by 14.4%, 12.3%, and 10.8%, respectively, in summer. The rate of the contribution of forest is 12.0%, and the contributions of other factors are between 6% and 10%.

**Figure 8:**
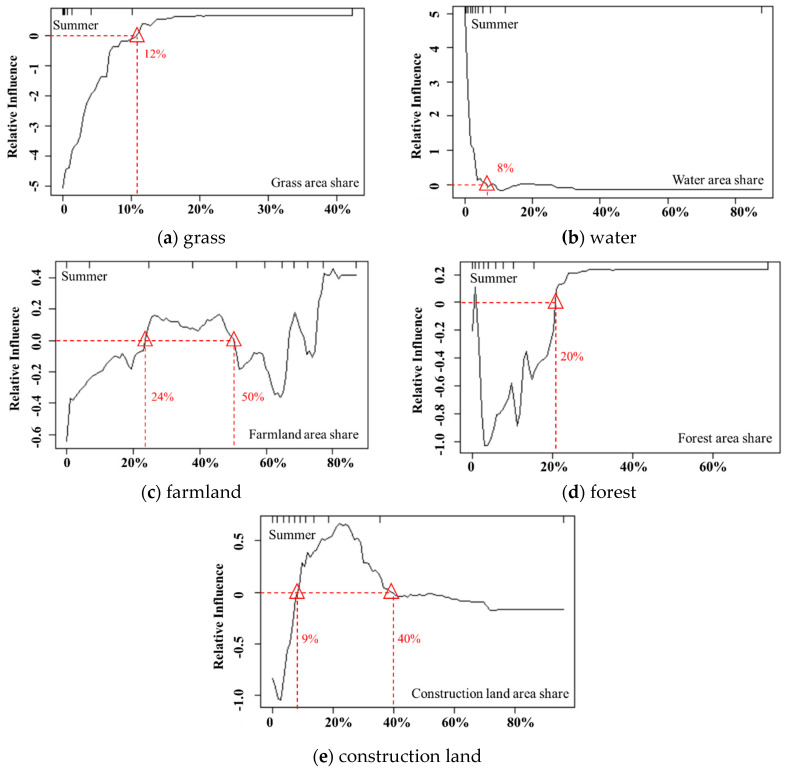
The influence of the main factors (concentration rate >10%) on PM_2.5_ in summer.

As illustrated by Figure 8, the influencing curve of the proportion of grassland area on PM_2.5_ in summer is similar to that in spring. The difference is that the negative effect values on PM_2.5_ in summer are strongly higher than those in spring, and the highest impact value in summer is twice that in spring, suggesting that the mitigation of PM_2.5_ by grassland becomes more significant in summer.

As in spring, the impact of water on PM_2.5_ in summer shows a gradual downward trend. When the water area is small, water has a positive impact on PM_2.5_. This impact is because the evaporation of water in summer leads to an increase in atmospheric humidity, which in turn makes it impossible for the air pollution flowing from adjacent land uses to diffuse. However, when the water area increases to 8–20%, water becomes weakly negatively correlated with PM_2.5_ and reaches its maximum value at the threshold of 10%. This result may be because the cold and wet effects of water bodies enable PM_2.5_ to absorb moisture and increase sedimentation, which effectively alleviates PM_2.5_ pollution [9]. This finding is similar to the research of Zhu et al. (2016) [34], who found that, when the water area reaches a certain area, the flow of cold and warm air between the water area and its surrounding areas becomes obvious, leading to the diffusion of air pollution matter to areas with low humidity, thus improving the air quality above the water body.

The impact of farmland on PM_2.5_ in summer exhibits a similar curve with that in spring, and the positive and negative effects on PM_2.5_ alternate. When the farmland area share varies between 24% and 50%, it has a positive impact on PM_2.5_, while farmland area shares greater than 50% have a strong negative correlation with PM_2.5_.

In summer, forest has a significant negative impact on PM_2.5_, with a V-shaped impact curve. As forest coverage increases, the negative impact on PM_2.5_ first increases and then decreases. When the forest coverage reaches 20%, the impact curve tends to be stable and stays near 0, indicating that in summer, a forest coverage of 20% can obtain an optimal PM_2.5_ mitigation effect, while excessive forest coverage has little effect on PM_2.5_.

The impact of construction land on PM_2.5_ in summer exhibits a similar curve with that in spring, first increasing and then decreasing. When the construction land area share in a region is greater than 9%, it has a significant positive effect on PM_2.5_. This pattern is closely related to the high intensity of human activities and large amounts of exhaust emissions. Moreover, construction lands are mostly impervious surfaces, which have a poor capacity to adsorb particulate matter; therefore, pollutants are more likely to diffuse into the air, thus increasing PM_2.5_ pollution.

#### 4.2.3. Explaining PM_2.5_ in Autumn

**Figure 9:**
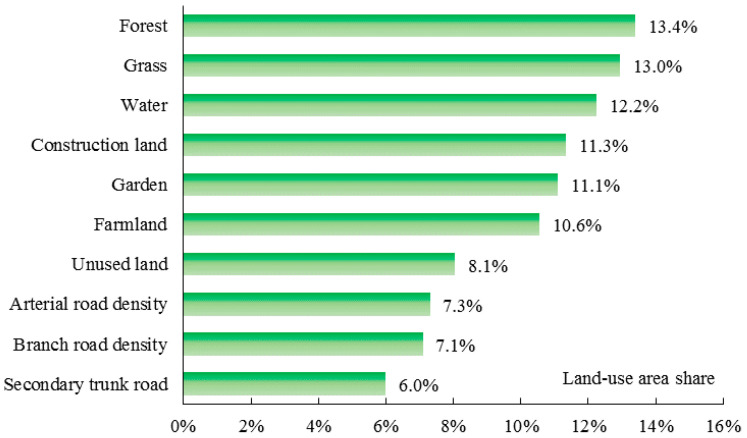
Relative impacts of each land-use distribution on PM_2.5_ in autumn.

Figure 9 illustrates the contributions (%) of land-use distribution on PM_2.5_ in autumn. Forest, with a contribution rate of 13.4%, was the dominant factor affecting PM_2.5_, followed by grass (13.0%). The contribution rates of water, construction land, gardens, and farmland to PM_2.5_ were 12.2%, 11.3%, 11.1%, and 10.6%, respectively. The contribution rates of other factors varied between 6% and 8.1%.

**Figure 10:**
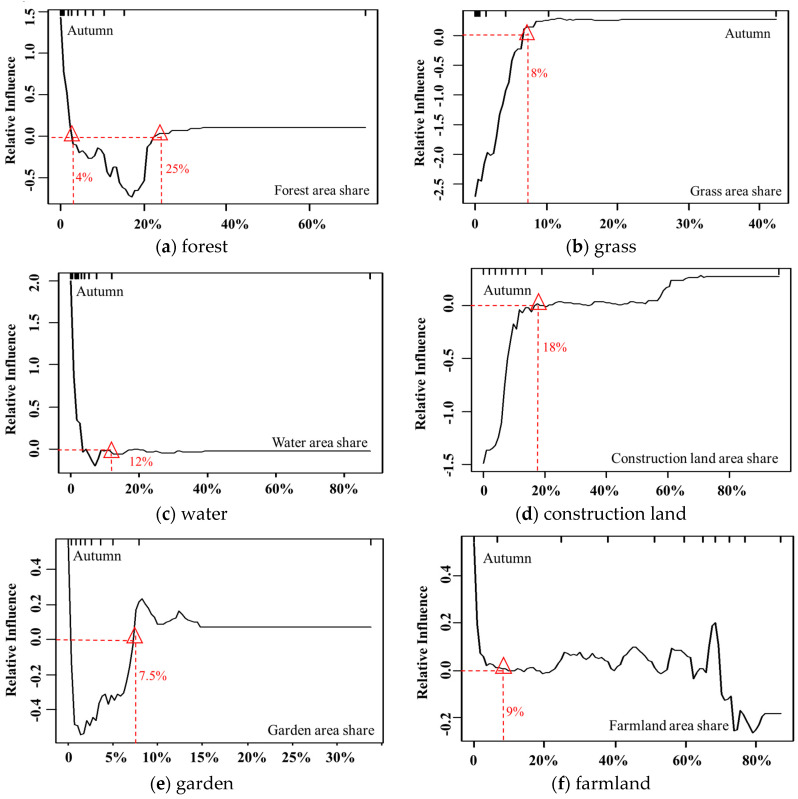
The influence of the main factors (concentration rate >10%) on PM_2.5_ in autumn.

As illustrated by Figure 10, as in spring and summer, forest is significantly negatively correlated with PM_2.5_, especially when the proportion is between 4% and 25%. Its negative impact on PM_2.5_ increases and reaches a maximum at the threshold of approximately 16% in autumn. When the forest area share is greater than 25%, the inhibitory effect on PM_2.5_ gradually decreases to zero.

The influence of grassland distribution on PM_2.5_ in autumn shows an upward curve, and the mitigation of PM_2.5_ is weaker than that in spring and summer. When the value is 0–8%, grassland has a negative effect on PM_2.5_, and as the grassland area increases, the negative impact decreases. When the grassland area reaches 8%, the impact on PM_2.5_ gradually becomes positive. This may due to that, as the weather becomes colder, most grass has withered, and the mitigative effect of grassland on PM_2.5_ will be greatly reduced, and thus as grass lands are surrounded mostly by construction lands, its impacts on PM_2.5_ concentrations may easily be affected by the discharge from surrounding artificial pollution.

The effects of water on PM_2.5_ show a downward trend with increasing water area. Similar to spring and summer, water has an overall positive influence on PM_2.5_. When the water area proportion is between 4% and 12%, water has a weak negative correlation with PM_2.5_, suggesting that as the weather cools, the inhibitory effect of water on PM_2.5_ increases slightly.

It should be noted that in autumn, the impact of construction land on PM_2.5_ shows a gradual upward trend, and its positive effect on PM_2.5_ becomes weaker than that in spring and summer. The reason may due to that in autumn, the weather becomes colder, the human activities decrease compared with spring and summer, thus gardens surrounding the construction lands may also help to mitigate pollution level. When the area of construction land is between 0% and 18%, it is negatively correlated with the PM_2.5_ concentration. Beyond this threshold, the impact becomes weakly positive.

Garden shows a V-shaped influencing curve, i.e., a trend of first decreasing and then increasing. When the garden area accounts for a proportion between 0.5% and 7.5%, it has a significant negative effect on PM_2.5_ and reaches its maximum at the threshold of 2%. The negative impact of gardens on PM_2.5_ is stronger in autumn than in spring.

The impact of farmland on PM_2.5_ in autumn alternates between being positive and negative. Farmland with an area share less than 9% has significant positive effects on PM_2.5_, which may be related to the fact that most of the farmland is bare land during rotation cultivation. With the increase in farmland area in the range between 40% and 70%, unlike in spring and summer, farmland has a significant positive impact on PM_2.5_, which may be due to the burning of wheat straw on the farmland in autumn, as suggested by Ding et al. (2013) [35]. When the farmland area exceeds 70%, it has a negative impact on PM_2.5_, indicating that when the farmland area exceeds a certain range, some farmland, such as winter wheat, can have a similar function as vegetation.

#### 4.2.4. Explaining PM_2.5_ in Winter

**Figure 11:**
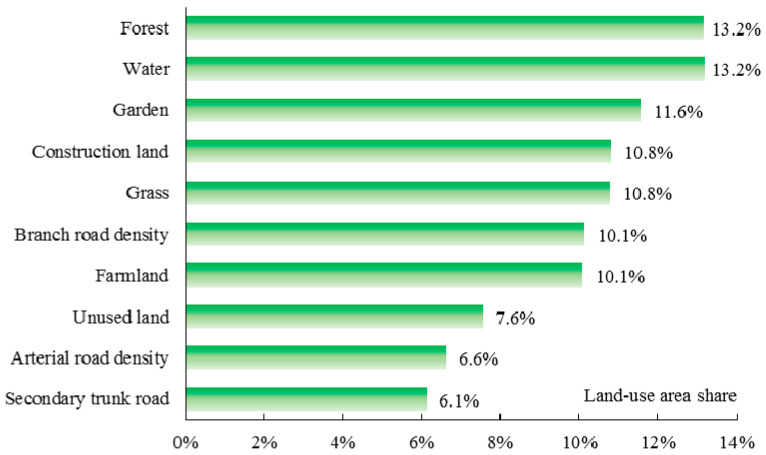
Relative impacts of each land-use distribution on PM_2.5_ in winter.

As illustrated in Figure 11, the first two dominant factors are forest and water, both with scores of 13.2%. The contribution rates of garden, construction land, grass, and farmland are 11.6%, 10.8%, 10.8%, and 10.1%, respectively. Different from the other three seasons, the branch road density has a higher contribution (10.1%) to PM_2.5_ in winter, indicating that the impact of roads on air quality in winter gradually increases. The contribution rates of the other factors range from 6% to 8%.

The impact of water on PM_2.5_ in winter shows a downward trend. When the proportion of water area is less than 22%, it has a positive impact on PM_2.5_. This result may be because as the weather becomes cold in winter, the cold and wet effect of water bodies enables PM_2.5_ to absorb moisture, thereby increasing sedimentation.

In contrast to the other three seasons, the impact of forest on PM_2.5_ in winter shows an overall upward trend. When the forest area proportion is less than 18%, it has a negative impact on the PM_2.5_ concentration. However, as the forest area increases, the negative impact gradually decreases and becomes weakly positively correlated with PM_2.5_. This result indicates that although forests can effectively alleviate PM_2.5_ pollution, as the weather becomes colder, most vegetation has withered, and the dust retention effect of forests will be greatly reduced compared with its role in spring and summer.

Unlike other seasons, gardens have a significant positive influence on PM_2.5_ in winter. There may be two reasons for this result. First, the withering of leaves on garden land in winter reduces the adsorption effect of PM_2.5_. Second, garden lands are surrounded mostly by residential and construction lands and are affected by artificial pollution sources, such as coal burning in winter [5,33], leading to severe PM_2.5_ pollution.

Construction land is one of the main factors influencing PM_2.5_ and is significantly positively correlated with PM_2.5_. Because the amount of construction land with a share more than 33% only accounts for 10%, the impact of construction land with an area proportion less than 33% was analyzed. The impact of construction land on PM_2.5_ in winter shows an overall upward trend, indicating that intensive population activities will aggravate PM_2.5_ pollution. Construction land has the greatest impact on PM_2.5_ at a spatial distribution of 25%.

The inhibitory effect of grassland on PM_2.5_ in winter is lower than that in the other three seasons. This result is because most grasslands gradually wither, and their ability to absorb air pollutants decreases as the weather turns colder. When the grassland area share is greater than 6%, grasslands are positively correlated with PM_2.5_. The threshold is decreased by 4% compared with that in the other three seasons, indicating that the impact of grassland on PM_2.5_ gradually decreases as the weather becomes colder. The reason for this pattern may be that grasslands are mostly distributed in urban built-up areas and are affected by coal heating in winter and human activities from these built-up areas; thus, the PM_2.5_ concentration in the grasslands may increase to some extent.

**Figure 12:**
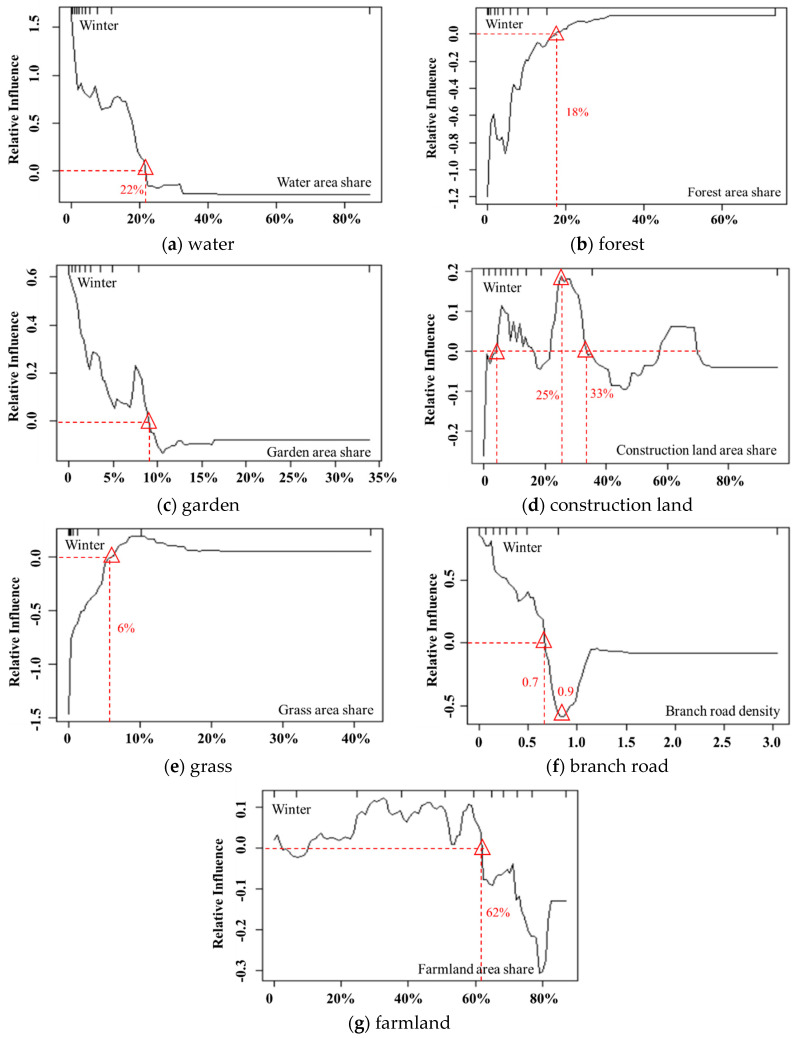
The influence of the main factors (concentration rate >10%) on PM_2.5_ in winter.

Branch roads are connecting channels between the trunk road and the road in each block, providing ease in resident transportation. In winter, the effect of the branch road density on PM_2.5_ increases significantly. Since the data on branch roads with a density of greater than 0.9 accounted for only 10% of all data, we analyzed only the case with a density less than 0.9. As illustrated in Figure 12, the impact curve of branch roads on PM_2.5_ decreases gradually in winter. This result means that as the branch road density increases, the positive impact of branch roads on PM_2.5_ gradually weakens. When the branch road density reaches 0.7, the positive impact decreases to 0. However, with a density greater than 0.9, the impact on PM_2.5_ is negative and reaches the maximum impact at a density of 0.9. This result is possibly because the increase in the branch road density causes the traffic flow to disperse and effectively avoids the accumulation of vehicle exhaust emissions, leading to the easy diffusion of pollutants.

The impact of farmland on PM_2.5_ in winter is similar to that in autumn. It is noteworthy that the positive effects in winter are more significant, probably for two reasons. First, this relationship is related to postharvest straw burning on farmland in winter. Second, most of the farmland is idle in winter, which is equivalent to bare land, and it is mostly adjacent to rural settlements and is thus influenced by coal heating in winter, leading to an increase in PM_2.5_ concentration [36].

### 4.3. Discussion and Policy Recommendations

Analyzing the impacts of land use on PM_2.5_ in different seasons is important for providing insights for urban planners to improve urban air quality. Understanding the influences may be critical for balancing limited financial resources for urban green space planning and PM_2.5_ mitigations. Based on the findings across different seasons, the following policy recommendations are provided.

First, we recommend strengthening the prevention of pollution in winter and cultivating more evergreen vegetation. Through the comprehensive analysis of seasonal differences in the impacts of land-use distribution on PM_2.5_, the findings showed that as temperature decreases, the contribution of green space (such as grassland and forest) to PM_2.5_ concentrations gradually decreases while the contribution of artificial structures increases. Specifically, as the weather gets colder, the contribution rate of grassland decreases from 16.6% in spring to 10.8% in winter. This pattern is mainly because, in cold weather, most grasslands and forests gradually wither, weakening the adsorption of air pollution matter by leaves, thus decreasing the mitigation effects on PM_2.5_. Therefore, the Weifang government should fully consider the seasonal differences in PM_2.5_ pollution, with limited urban green areas, and more efforts should be made to strengthen the cultivation of cold-season lawns and evergreen vegetation. Considering the climate characteristics of Weifang and the resistance effect of vegetation on atmospheric particulates [37], it is recommended that Weifang city should strengthen the planting of evergreen trees (such as *Pinus bungeana* Zucc., *Pinus tabulaeformis* Carr.), evergreen shrub (such as *Euonymus japonicus*) [38], and *Lolium perenne*. In addition, as a special land-use type of three-dimensional green space, the distribution of farmland has an alternative effect on PM_2.5_, specially, in spring, summer, and autumn, farmland can effectively reduce PM_2.5_ concentrations through the dry and wet sedimentation of leaves, i.e., in the same manner as regular vegetation. However, it is noteworthy that the positive effects of farmland on PM_2.5_ are strengthened in winter, which is closely related to straw burning after harvesting in winter. Therefore, relevant policies, such as improving the industrial utilization of straw, constructing straw collection, storage, and transportation system, should be formulated to control straw burning in rural areas in winter. The impacts of garden on PM_2.5_ also has seasonal characteristics like farmland, when the garden area share is less than a certain threshold in spring, summer, and autumn, gardens can effectively suppress PM_2.5_ pollution, while when below this threshold in winter, gardens can obviously aggravate regional pollution. Construction land and roads can significantly increase PM_2.5_ in Weifang, and as the weather gets colder, their impacts on PM_2.5_ increase and peak in winter. This result may be because the low temperature and weak convection in winter do not favor the diffusion of pollution particles produced by human activities, such as coal heating and traffic exhaust, leading to an increase in PM_2.5_ in the region. Therefore, this paper recommends that the Weifang government should promote the prevention and control of pollution in winter and plant more evergreen vegetation in urban street park, traffic circles, and other open spaces to effectively reduce PM_2.5_ pollution in winter.

Second, optimal land-use distribution planning should be quantitatively formulated. The BRT model reflects the dynamic contribution rates of land-use factors to PM_2.5_ in different seasons. Understanding these quantified contributions is particularly important for balancing limited green spaces and financial resources to minimize PM_2.5_ pollution. By capturing the turning points of the impact curves of vegetation (such as grassland and forest), this paper found that when the vegetation area in the region reaches a certain proportion, its inhibitory effect on PM_2.5_ tends to be stable rather than infinitely increasing. Urban planners can consider different scenarios for alleviating the effects of PM_2.5_ by balancing the spatial distribution of construction and green spaces: Scenario A: The construction land area share <11%, and there is no need to control the vegetation coverage in the region due to the low impact of construction land on PM_2.5_; scenario B: The construction land area share >11%, in these areas, measures and implications should be taken to alleviate PM_2.5_ pollution in the region. For instance, green vegetation should be planted, but it should be noted that there is no need to overplant green vegetation. It is recommended to plant grass and forest, which area shares shall be approximately 8% and 20%, respectively, in the panning area, in order to mitigate air pollution maximum. Additionally, as the conditions permit, increasing the width of branches connecting different main roads, or constructing more branches connecting main roads, so as to ensure that the regional branch road density is greater than 0.7, which can avoid the accumulation effect of vehicle exhaust emissions and to improve air quality. The above findings and implications can be used as important references for urban land-use planning.

Finally, the simulation of PM_2.5_ pollution in different situations can be performed on the micro scale. Urban planners can perform simulations on the impact of land-use distribution on regional PM_2.5_ in different scenarios to provide theoretical guidance for land-use planning under a micro grid scale by using the BRT model. First, the region to be planned should be divided into a micro grid space with 5 km cells. Then, let X_k_ be the vector of 10 land-use area shares, and the land-use plan can be expressed as the vector X = (X_1_, X_2_…X_k_…X_n_), where *n* is the total number of grid cells. Finally, the mapping from X to PM_2.5_ can be done with the estimated BRT model, PM_2.5_ = BRT(X). Urban planners can then simulate in detail the distribution of the predicted PM_2.5_ concentrations in different scenarios of land-use distribution on the micro grid scale.

## 5. Conclusions

This paper introduced the BRT model to investigate the various contributions of different land-use distributions to PM_2.5_ across four seasons in Weifang. The main conclusions are as follows.

(1) The PM_2.5_ pollution is high in winter and low in summer. Grassland, forest, water, branch road, and construction land do not show seasonal characteristics, the former two factors can significantly inhibit PM_2.5_ pollution when below a threshold; however, the construction land can intensify PM_2.5_ pollution when greater than a threshold. Garden and farmland have seasonal characteristics. The spatial distributions of other land uses have negligible impacts on regional PM_2.5_.
(2) The dominant factor affecting PM_2.5_ in spring and summer is grassland distribution and that in autumn and winter is the forest distribution. As the weather becomes colder, the impacts of the grassland and forest on PM_2.5_ gradually decrease, whereas the opposite pattern was found for artificial lands.
(3) According to the findings, it is recommended to strengthen pollution prevention in winter and plant more evergreen vegetation in urban open space areas.

This study is of great significance for future research in terms of providing an in-depth understanding of the heterogeneity and seasonal variations in the impact of land-use distributions on PM_2.5_, and the findings and policy recommendations can contribute to policy-making aimed at PM_2.5_ pollution mitigation for Weifang city and other similar cities all over the world [39]. At the same time, it proposes a general framework different from the existing research, which focused on not only qualitative determination of the negative or positive impact of each factor on PM_2.5_ but also to some extent reflect the size of the impact. However, this study also has limitations. For instance, this paper used the IDW interpolation for data integration, which may lead to uncertainty results; other interpolation methods like the interpolation algorithm that consider both spatial and temporal characteristics, and the hybrid kriging method [40] could be considered. Moreover, the precise quantitative and mutual effects for factors need further study, such as integrating models like the spatial autocorrelation model with BRT. Finally, future research will also involve the performance comparison of BRT with other learning models, and multiple time series analyses will be included to further explore the spatiotemporal differences of these impacts.
